# PExFInS: An Integrative Post-GWAS Explorer for Functional Indels and SNPs

**DOI:** 10.1038/srep17302

**Published:** 2015-11-27

**Authors:** Zhongshan Cheng, Hin Chu, Yanhui Fan, Cun Li, You-Qiang Song, Jie Zhou, Kwok-Yung Yuen

**Affiliations:** 1 Department of Microbiology, The University of Hong Kong, Pokfulam Road, Hong Kong; 2 State Key Laboratory of Emerging Infectious Diseases, The University of Hong Kong, Pokfulam Road, Hong Kong; 3 Research Centre of Infection and Immunology, The University of Hong Kong, Pokfulam Road, Hong Kong; 4 Carol Yu Centre for Infection, The University of Hong Kong, Pokfulam Road, Hong Kong; 5 Department of Biochemistry, The University of Hong Kong, Pokfulam Road, Hong Kong; 6 Center for Genomic Sciences, The University of Hong Kong, Hong Kong Special Administrative Region, China

## Abstract

Expression quantitative trait loci (eQTLs) mapping and linkage disequilibrium (LD) analysis have been widely employed to interpret findings of genome-wide association studies (GWAS). With the availability of deep sequencing data of 423 lymphoblastoid cell lines (LCLs) from six global populations and the microarray expression data, we performed eQTL analysis, identified more than 228 K SNP cis-eQTLs and 21 K indel cis-eQTLs and generated a LCL cis-eQTL database. We demonstrate that the percentages of population-shared and population-specific cis-eQTLs are comparable; while indel cis-eQTLs in the population-specific subsection make more contribution to gene expression variations than those in the population-shared subsection. We found cis-eQTLs, especially the population-shared cis-eQTLs are significantly enriched toward transcription start site. Moreover, the National Human Genome Research Institute cataloged GWAS SNPs are enriched for LCL cis-eQTLs. Specifically, 32.8% GWAS SNPs are LCL cis-eQTLs, among which 12.5% can be tagged by indel cis-eQTLs, suggesting the fundamental contribution of indel cis-eQTLs to GWAS association signals. To search for functional indels and SNPs tagging GWAS SNPs, a pipeline Post-GWAS Explorer for Functional Indels and SNPs (PExFInS) has been developed, integrating LD analysis, functional annotation from public databases, cis-eQTL mapping with our LCL cis-eQTL database and other published cis-eQTL datasets.

More than ten thousands single nucleotide polymorphisms (SNPs) have been identified to associate with complex traits and human diseases in genome-wide association studies (GWAS) in the past decade^1^. Since most of the GWAS significant SNPs are located in non-coding or intergenic regions, the molecular mechanism underlying the association or the causal gene cannot be directly inferred from the SNPs. On the other hand, a typical GWAS may yield plenty of significant SNPs. It would be highly desirable if functional relevance of GWAS significant SNPs could be obtained from public databases and candidate variants could be prioritized for validation. With next generation sequencing (NGS) data of the 1000 Genomes (1 KG) Project available to the scientific community, it is now feasible to have a more in-depth interpretation of the GWAS association signals by utilizing the 1 KG data to visualize the linkage disequilibrium (LD) patterns of GWAS SNPs with other variants within the human genome^2^.

The 1 KG data of phase 1 release presents an extensive catalog of human variations including 38.2 M SNPs, 3.9 M short indels and 14 K deletions in 1,092 individuals from 14 global populations. The latest phase 3 release expands the phase 1 release to include 2,504 individuals from 27 global populations. When a specific SNP with unknown functional implication is identified in a GWAS, the functional variant(s) could be potentially pinpointed based on the LD context of the SNP observed in the 1 KG data and the functional annotations such as Ensembl regulatory features generated by Ensembl project^3^. The Ensembl project has generated an expanding wealth of information including, but not limited to, gene structure, genetic variations and their consequences as well as functional genomic data. These comprehensive databases have provided the most abundant resource to functionally interpret the genetic variations in human genome. The variants that are in high LD with GWAS SNPs may be mapped to putative regulatory regions defined in Ensembl Regulatory Build^3^, from which the functional implication of GWAS SNPs could be postulated.

Currently, a number of tools, such as SNAP^4^ and LocusZoom^5^ can generate LD plot for GWAS SNPs and their high LD SNPs. However, the LD pattern between SNPs and structure variants including small insertion/deletion (<50 bp) and large insertion/deletion (>1 kb) (both referred to as indel afterwards) have not been extensively examined. Indels are the second abundant type of genetic variations in human genome. It has been suggested that indels contribute substantially to both inherited traits and human diseases^6^, since they may give rise to more severe functional alterations in the coding regions, as well as 5′- and 3′-UTR regions in comparison with SNPs^7^,^8^,^9^,^10^,^11^. Therefore, interrogating indels in GWAS is acutely needed.

Another unexplored area for indels is the expression Quantitative Trait Loci (eQTL) mapping. To date, eQTL studies in human cells and tissues have resulted in the identification of thousands of “cis-eQTLs” and “trans-eQTLs”^12^,^13^, which are referred to genomic loci correlate to mRNA expression levels of a specific gene in cis (locally) and in trans (at a distance), respectively. With the systematically generated eQTL data, a significant SNP could be potentially translated into an eQTL for specific gene(s). Consequently, the putative causal gene can be pinpointed for further functional validation. Although extensive efforts have been devoted to identify SNP eQTLs (also known as expression SNP, eSNP)^14^,^15^,^16^,^17^,^18^, indel eQTLs have not been explored genome-wide due to the difficulty in discovery of indels with genotyping methods for SNPs^19^. The availability of NGS data of lymphoblastoid cell lines (LCLs) has enabled the systematic interrogation of indels and the identification of indel eQTLs. Additionally, the SNP eQTLs in LCLs can be revealed at a higher resolution.

In this study, an integrative approach was utilized to identify SNP cis-eQTLs and indel cis-eQTLs in 423 LCLs from six global populations. We assembled all the cis-eQTLs as well as their functional information and generated a LCL cis-eQTL database. We characterized the LCL SNP cis-eQTLs and indel cis-eQTLs in genomic context. We tested the enrichment of cis-eQTLs proximate to the transcription start site (TSS). We demonstrated that LCL cis-eQTLs represent a substantial proportion of the National Human Genome Research Institute (NHGRI) cataloged GWAS SNPs. Notably, in order to facilitate the searching of functional variants, especially previously unexplored indels in high LD with user-interested SNPs, we developed a pipeline named as Post-GWAS Explorer for Functional Indels and SNPs (PExFInS). Finally, we demonstrated the application of PExFInS to pinpoint causal variants for human diseases.

## Results

### cis-eQTL distribution and sharing among populations

We investigated the distribution and sharing of cis-eQTLs among six global populations. The cis-eQTL analysis was performed in 423 LCLs, including 73 CEU (Utah residents with northern and western European ancestry), 77 CHB (Han Chinese in Beijing, China), 72 JPT (Japanese in Tokyo, Japan), 80 LWK (Luhya in Webuye, Kenya), 42 MEX (Mexican ancestry in Los Angeles) and 79 YRI (Yoruba in Ibadan, Nigeria). cis-eQTLs were mapped by correlating gene expression of 14,010 unique autosomal RefSeq genes to the genotypes of 1 KG variants of phase 1 release. These variants are located in cis (200 kb upstream and downstream) to the RefSeq genes. At cis-eQTL cutoff *P* value < 10^−4^, there are a total of 21,841 indel cis-eQTLs and 228,743 SNP cis-eQTLs in at least one population (Supplementary Fig. 1). The number of shared indel cis-eQTLs and SNP cis-eQTLs in at least two populations are 4,976 (22.8%) and 53,926 (23.6%), respectively. The cis-eQTLs exclusively shared by two populations were also determined. The largest pairwise sharing in only two populations is observed between CHB and JPT with 1,040 indel cis-eQTLs and 11,334 SNP cis-eQTLs. It is around 7-fold greater than those of pairwise sharing in CHB with each of the other four populations, including CEU, YRI, LWK and MEX. The result is consistent with a previous study^13^ demonstrating that closely-related populations CHB and JPT share more cis-eQTLs than more distantly-related populations. The second largest pairwise sharing in only two populations is uncovered in other two closely-related populations YRI and LWK^20^, with 501 indel cis-eQTLs and 5,201 SNP cis-eQTLs.

We further partitioned both SNP cis-eQTLs and indel cis-eQTLs into two categories, population-specific and population-shared. The cis-eQTLs present in at least two populations and those present in one population exclusively are defined as population-shared and population-specific, respectively. We compared the percentage of population-shared cis-eQTLs with that of population-specific cis-eQTLs in six populations using Student’s t test. As shown in Table 1, the percentage of population-shared cis-eQTLs (46.8 ± 6.4%) is comparable to that of population-specific cis-eQTLs (53.18 ± 6.39%; *P* = 0.112). However, the relatively ancient populations, including YRI and LWK, tend to have less population-shared SNP cis-eQTLs and indel cis-eQTLs with the other 5 populations. Interestingly, the ratios of indel cis-eQTLs versus SNP cis-eQTLs in population-specific cis-eQTLs (0.097 ± 0.003, mean ± SD) are significantly higher than those in population-shared cis-eQTLs (0.091 ± 0.003, Student’s t test *P* = 6.90 × 10^−3^). To exclude the possibility that the higher ratio of indel cis-eQTLs versus SNP cis-eQTLs in population-specific subsection may be attributed to the existence of more high LD indels than SNPs, we re-generated the LCL cis-eQTLs by keeping only the peak indel cis-eQTL and SNP cis-eQTL for each gene probe. Subsequently, we re-calculated the ratio of indel cis-eQTLs versus SNP cis-eQTLs. It was observed that the ratios remained significantly higher in the population-specific subsection (0.109 ± 0.005) than in population-shared subsection (0.096 ± 0.005, *P* = 6.00 × 10^−4^). Notably, the differences between population-specific and population-shared subsection are enlarged in all populations after excluding the possible contribution of LD effect. Therefore, there exists a modest but consistent enrichment of indel cis-eQTLs over SNP cis-eQTLs in population-specific subsection among all populations, suggesting that indel cis-eQTLs in the population-specific subsection account for a greater proportion of gene expression variations than the counterparts in population-shared subsection.

**Figure 1:**
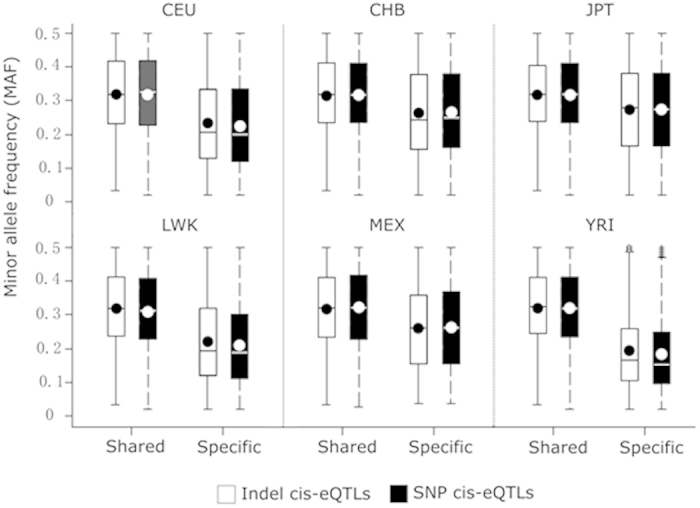
Minor allele frequencies of lymphoblastoid cell line (LCL) cis-eQTLs. The minor allele frequencies of LCL cis-eQTLs (cutoff *P* value < 10^−4^) are illustrated by grouping cis-eQTLs into four categories, including population-specific SNP cis-eQTLs, population-shared SNP cis-eQTLs, population-specific indel cis-eQTLs, and population-shared indel cis-eQTLs. cis-eQTLs shared in at least two populations are assigned as population-shared cis-eQTLs, or population-specific ones otherwise. The boxes represent the interquartile range. The lines and dots within these boxes define the median and mean, respectively. In the YRI population, open dots and plus signs are possible outliers.

The minor allele frequencies (MAFs) of indel cis-eQTLs and SNP cis-eQTLs are comparable in each population (Fig. 1). However, in all populations, the MAFs of population-shared cis-eQTLs, including indel cis-eQTLs and SNP cis-eQTLs, are significantly higher than those of population-specific cis-eQTLs (Student’s t test, all *P* values < 10^−4^). Furthermore, cis-eQTLs specific in YRI, an ancient population, show relatively lower and more variable MAF than the specific cis-eQTLs in other populations. Taken together, we demonstrated that population genetic relatedness is an important determinant for the sharing of cis-eQTLs among populations. The percentages of population-shared and population-specific cis-eQTLs in the six populations are comparable while indel cis-eQTLs in population-specific subsection make more contribution to gene expression variations than those in population-shared subsection. Additionally, SNP cis-eQTLs and indel cis-eQTLs have comparable MAFs in most populations.

### Genomic properties of indel cis-eQTLs and SNP cis-eQTLs

**Figure 2:**
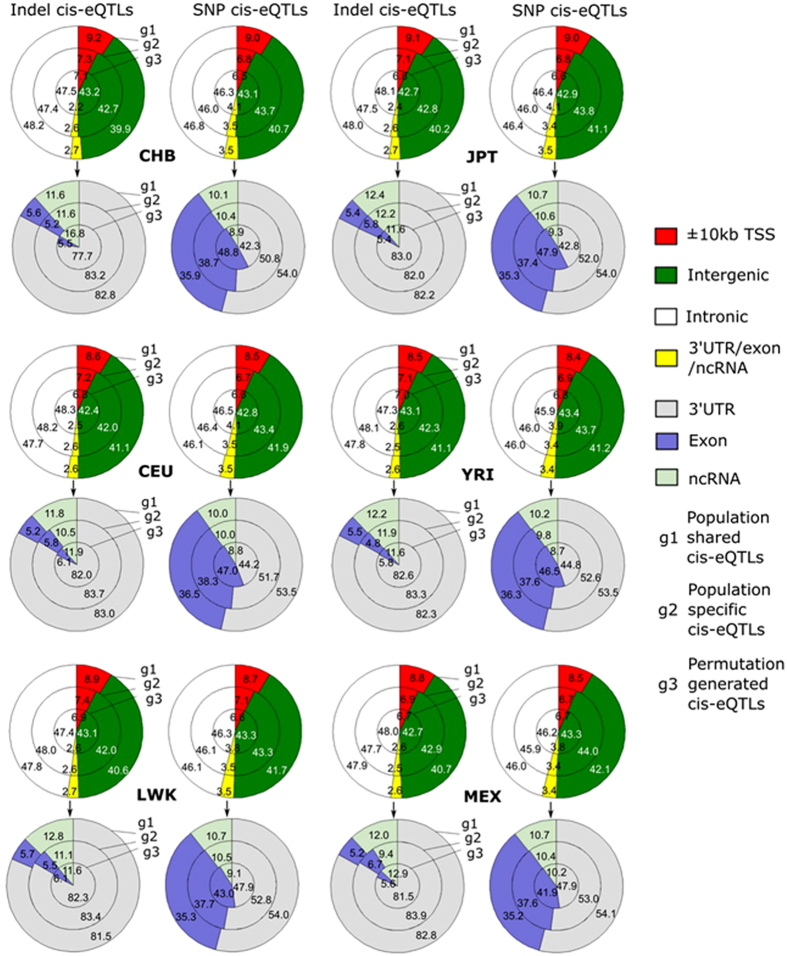
Distribution of cis-eQTLs and fake cis-eQTLs in various genomic regions. cis-eQTLs were generated in six global populations, including CHB, JPT, CEU, YRI, LWK and MEX. Fake cis-eQTLs in the above six populations were generated by 100 times of genome-wide permutation. All cis-eQTLs and fake cis-eQTLs are partitioned into indel cis-eQTLs and SNP cis-eQTLs. cis-eQTLs are further classified into population-shared and population-specific cis-eQTLs. All cis-eQTLs and fake cis-eQTLs are mapped to various genomic regions, including region ± 10 kb relative to the transcription start site (±10 kb TSS), intron, intergenic region, exon, non-coding RNA (ncRNA), and 3′ untranslated region (3′UTR), the percentages of which are illustrated within each piechart. Since cis-eQTLs located in 3′UTR, exon and ncRNA individually account for a small percentages of total cis-eQTLs, they are grouped together as 3′UTR/exon/ncRNA in the upper panel. The latter is further zoomed in to illustrate the percentage of cis-eQTLs mapped to each individual location. The percentages of population-shared (g1) and population-specific (g2) cis-eQTLs along with the fake cis-eQTLs (g3) are illustrated in the piecharts. Chi-squared test was used for the data analysis.

**Figure 3:**
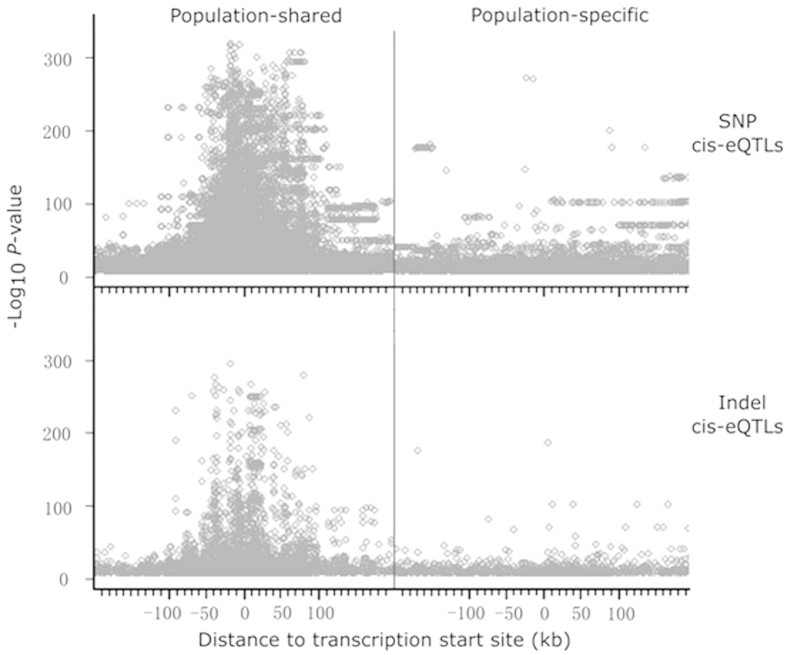
Distribution of cis-eQTLs relative to the transcription start site. SNP cis-eQTLs and indel cis-eQTLs are partitioned into population-specific and population-shared ones. The distribution pattern of cis-eQTLs relative to the transcription start site (TSS) is illustrated. The x-axis represents the distance of cis-eQTLs relative to TSS, while the y-axis represents the log_10_(*P* value) of cis-eQTLs. cis-eQTLs that pass the cutoff *P* value <10^−8^ are illustrated.

We assessed the genomic distribution of LCL cis-eQTLs (*P* value < 10^−4^) and compared these cis-eQTLs with fake cis-eQTLs generated by 100 times of genome-wide permutation which aims to have a distribution of cis-eQTLs under null hypothesis of no true association. Each permutation generated 1,082 ± 329 (average ± SD) indel cis-eQTLs and 18,906 ± 9,066 SNP cis-eQTLs in each population. We grouped SNP and indel cis-eQTLs into population-shared and population-specific cis-eQTLs. We compared the distribution pattern of population-shared and population-specific indel cis-eQTLs and SNP cis-eQTLs with their counterparts of permutation generated fake cis-eQTLs in various genomic regions, including ±10 kb to TSS, intron, intergenic region, exon, non-coding RNA (ncRNA), and 3′ untranslated region (3′UTR). At the cutoff *P* value < 10^−4^, the percentages of SNP cis-eQTLs and indel cis-eQTLs within ± 10 kb to TSS, especially the population-shared ones, are invariably and significantly higher than those of the fake cis-eQTLs in the six populations (Fig. 2, chi-squared test, all *P* < 10^−4^). Specifically, around 9% of population-shared indel cis-eQTLs and SNP cis-eQTLs are located within ± 10 kb to TSS while the percentages of population-specific cis-eQTLs in this region are around 7%, both significantly higher than those of fake cis-eQTLs. Further examination of cis-association signals of population-shared and population-specific cis-eQTLs revealed that most population-shared cis-eQTLs, including indel cis-eQTLs and SNP cis-eQTLs, display higher cis-association signals and distribute symmetrically toward TSS (Fig. 3). Interestingly, in all populations, SNP cis-eQTLs are significantly depleted from the exonic region (Fig. 2, chi-squared test, all *P* < 10^−4^). Additionally, only SNP cis-eQTLs but not indel cis-eQTLs are enriched in 3′UTR (Fig. 2, all *P* < 10^−4^). Taken together, we demonstrate that population-specific cis-eQTLs and population-shared cis-eQTLs are enriched in the region proximate to TSS, among which population-shared SNP cis-eQTLs and indel cis-eQTLs, display more intensive enrichment than the population-specific counterparts towards TSS region.

### LCL cis-eQTLs and GWAS SNPs

Previous studies have demonstrated that GWAS association SNPs are significantly enriched for SNP cis-eQTLs^21^. We searched the GWAS association SNPs in NHGRI catalog (released on Feb 10, 2015) in our LCL cis-eQTL database. Among 14,718 GWAS SNPs with unique dbSNP rs IDs, a total of 14,172 SNPs (96.3%) have genotyping data in the 1 KG data of phase 1 release and are associated with 1,099 diseases and traits. At the cis-eQTL cutoff *P* value < 10^−2^, 4,643 out of 14,172 (32.76%) GWAS SNPs are LCL SNP cis-eQTLs (GWAS eSNPs) for 4,378 genes in at least one population. These 4,643 GWAS eSNPs are associated with 860 (78.25%) out of 1,099 diseases and traits, including asthma, allergic rhinitis, bipolar disorder, breast cancer, cervical cancer, blood pressure, body mass index, inflammatory bowel disease, etc.

In current GWAS, SNPs but not indels are interrogated. Therefore, it is unknown to what extent indel cis-eQTLs can tag the GWAS association signals. It was suggested that indels cis-eQTLs are more likely to be functional than SNPs cis-eQTLs^22^,^23^. Thus, we searched for indel cis-eQTLs in high LD with these GWAS eSNPs. At the cis-eQTL cutoff *P* value < 10^−2^, 1,282 indel cis-eQTLs are in high LD (r^2^ ≥ 0.7) with 1,007 SNP cis-eQTLs (21.7.6%) out of 4,643 GWAS eSNPs. A total of 688 indel cis-eQTLs display perfect LD (r^2^ = 1) with 582 SNP cis-eQTLs in at least one population, which accounts for 12.5% of all GWAS eSNPs.

Taken together, we demonstrated that SNP and indel cis-eQTLs are significantly enriched around TSS, where population-shared cis-eQTLs display more intensive enrichment than population-specific cis-eQTLs. Furthermore, around one third of the NHGRI cataloged GWAS SNPs are cis-eQTLs in our LCL cis-eQTLs database, which are associated with more than three quarters of investigated human diseases and traits. A substantial proportion of these GWAS eSNPs can be tagged by indel cis-eQTLs, suggesting that indel cis-eQTLs may fundamentally contribute to the GWAS association signals.

### PExFInS: A pipeline searching for functional variants

Based on the above findings, cis-eQTLs, especially the previously neglected indel cis-eQTLs, are literally important taggers for GWAS SNPs. Therefore, integrating SNP cis-eQTLs and indel cis-eQTLs with GWAS SNPs could facilitate the identification of causal variants or causal genes. Meanwhile, the functional information encoded by GWAS SNPs and their taggers, such as regulatory features from the Ensembl regulatory database, will be conducive to the discovery of functional or causal variant(s) for human diseases. We developed a pipeline, PExFInS to integrate the newly-generated LCL cis-eQTL data, as well as publicly available cis-eQTL datasets derived from lung tissues^24^, liver tissues^14^, human monocytes^25^, dendritic cells^26^, and blood^27^. Additionally, splicing cis-eQTLs and protein cis-eQTLs generated in LCLs^28^,^29^ have also been incorporated into the pipeline.

**Figure 4:**
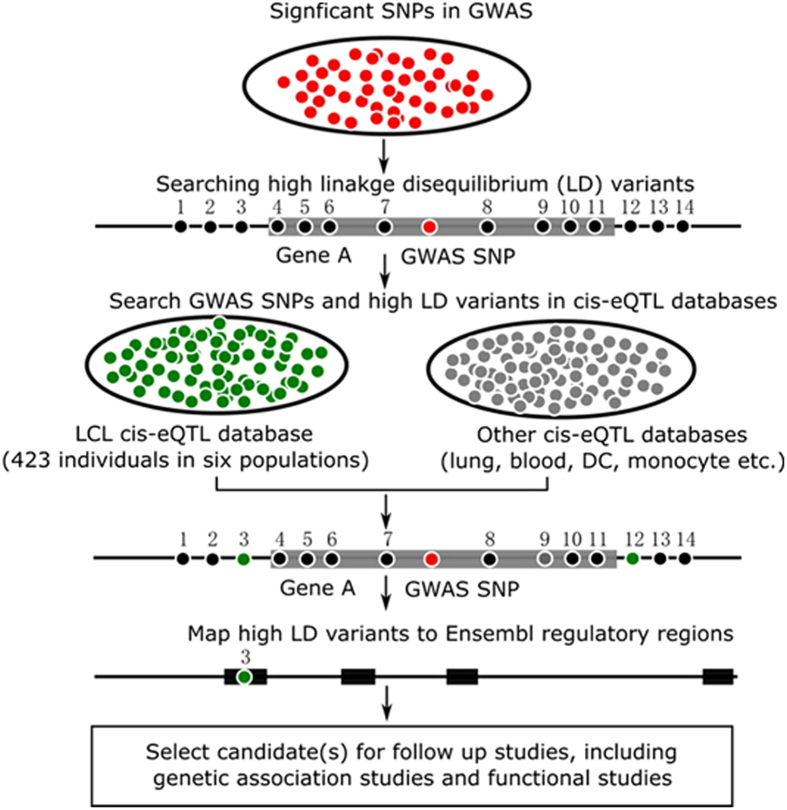
The strategy utilized by PExFInS in searching for functional variants. Three categories of analysis are implemented in PExFInS. Firstly, a pool of significant SNPs generated from GWAS are input to search for high linkage disequilibrium (LD) variants (determined by r^2^) based on the genotyping data of the 1000 Genomes Project (1,092 individuals in phase 1 release or 2,504 individuals in phase 3 release). Secondly, all the high LD variants are applied to cis-eQTL mapping with the lymphoblastoid cell line (LCL) cis-eQTL database and other published cis-eQTL data. Thirdly, these high LD variants are mapped to the Ensembl regulatory regions. Potential functional variant, such as variant 3 in the figure, can be selected as a candidate for further study, including genetic association study for replication and functional validation.

PExFInS can be utilized to search for high LD variants, including SNPs and previously unexplored indels, with user-queried SNPs using the genotyping data from the 1 KG data of phase 1 release (1,092 individuals) or the latest phase 3 release (2,504 individuals). The high LD variants are then mapped to cis-eQTLs and Ensembl regulatory regions, thereby the functional relevance of user-queried SNPs can possibly be revealed. The strategy is illustrated in Fig. 4. The high LD variant 3 localized in an Ensembl regulatory region and also a LCL cis-eQTL can be prioritized as a potentially functional variant, which can tag the GWAS SNP of interest and be brought forward for replication in another cohort. Additionally, the gene whose expression is correlated with genotypes of variant 3 can be applied for functional validation in molecular biology or cellular biology studies.

**Figure 5:**
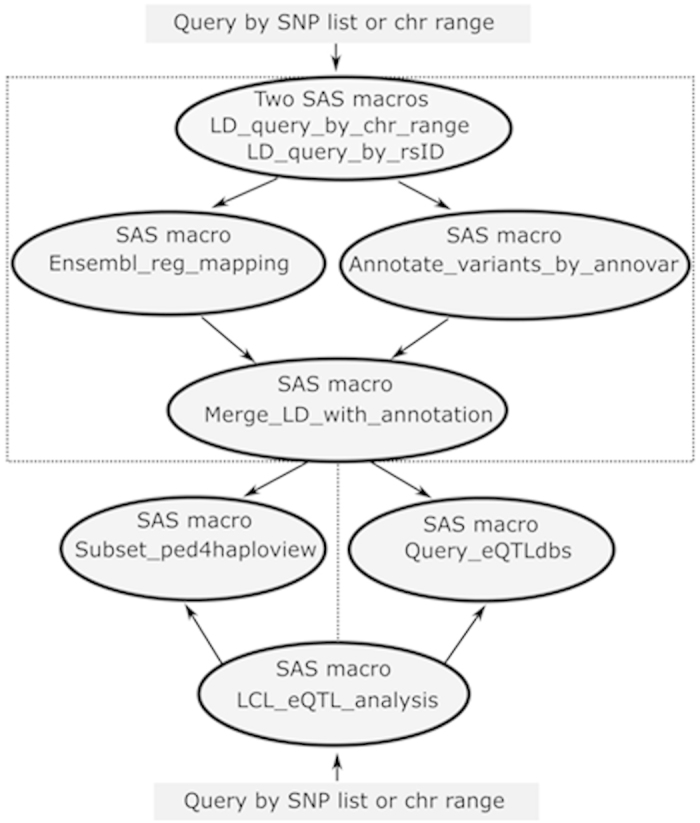
Implementation of PExFInS with SAS statistical language. Seven SAS macros are included in PExFInS. These macros can run along or together. User only needs to provide SNP list (dbSNP rs IDs) or the chromosome range (human genome build hg19) to calculate the pairwise linkage disequilibrium (LD) values (r^2^ and D’) and retrieve all high LD variants. These high LD variants can be further annotated. User may also query expression Quantitative Trait Loci (eQTL) databases or create LD plots. cis-eQTL and trans-eQTL analysis with 423 lymphoblastoid cell lines (LCLs) from six global populations are also implemented in PExFInS.

PExFInS is written in SAS statistical language (Fig. 5). A total of seven SAS macros are created to perform LD analysis, eQTL analysis, and regulatory feature mapping. These macros can work along or in combination. With the input dbSNP rs IDs or specific chromosome range (human genome build hg19), PExFInS is able to output all high LD variants as well as the functional annotations and eQTL data. Additionally, PExFInS can provide genotyping data required by Haploview^30^ to plot the LD for input variants and the high LD variants. For example, if users are interested in the LD pattern among candidate variants and GWAS SNPs, PExFInS can retrieve genotypes of all these variants and define their LD and haplotypes. Furthermore, PExFInS utilizes a powerful annotation tool, ANNOVAR^31^, to annotate the input SNPs and LD-derived variants with annotation databases from the UCSC Genome Browser^32^ and map these variants to RefSeq genes, conserved regions, transcription factor binding sites, and DNase hypertensive sites. The regulatory features of these variants from the Ensembl Regulatory Build can also be mapped and further combined with ANNOVAR annotations. Finally, all the functional information, especially cis-eQTL information and Ensembl regulatory features, can be included in a customized track for visualization in UCSC Genome Browser.

### Application of PExFInS

A promoter SNP, rs2564978, of the *CD55* gene is used to demonstrate the application of PExFInS. Our previous study revealed that rs2564978 was associated with the disease severity of the 2009 A(H1N1) pandemic influenza in the Chinese population^33^. PExFInS was used to search for functional variants that are in high LD with rs2564978 from the 1 KG data of phase 1 release. Within 1 kb upstream or downstream of the *CD55* gene, 40 variants are in tight LD with rs2564978 (r^2^ > 0.80) in 846 individuals from the populations of East Asian (ASN), Ad Mixed American (AMR) and European (EUR). The similar LD pattern is observed when the three super populations are tested individually (data not shown). An indel rs150046210 (synonyms rs3841376), is in high LD with rs2564978 among these three super populations (r^2^ > 0.90). The strong LD between the two variants was verified when the genomic DNA samples from 50 local Chinese were examined. It is shown that rs2564978 is literally in prefect LD with rs150046210 in homozygous carriers (r^2^ = 1), where rs2564978 C/C and T/T genotypes unequivocally coexist with rs150046210 insertion and deletion, respectively^33^. According to ANNOVAR annotation, rs150046210 is located in a DNase hypersensitive region and transcription factor binding cluster for Pol II and STAT3, indicating that the indel may regulate *CD55* mRNA transcription. In addition, PExFInS mapped the indel to an Ensembl regulatory region, ENSR00000551839. Moreover, in our LCL eQTL analysis, rs150046210 is significantly correlated to the gene expression of *CD55* in CHB LCLs (recessive model, *P* = 0.024). We performed the *in vitro* experiments to verify the regulatory effect of rs150046210. The reporter gene luciferase assay result showed that rs150046210, but not rs2564978, functionally regulates *CD55* transcription in a genotype-specific manner^33^.

Additionally, we analyzed a SNP rs12628403 that is associated with the risk to breast cancer^34^ using PExFInS. We uncovered that an indel (~30 kb), esv2666691, is in high LD with rs12628403 in the ASN (r^2^ = 0.89), AMR (r^2^ = 0.95) and EUR (r^2^ = 0.95) populations. PExFInS revealed that the indel is a LCL cis-eQTL of *APOBEC3B* gene in CHB (genotypic *P* value 5.1 × 10^−106^), JPT (1.1 × 10^−164^), CEU (1.2 × 10^−14^), and MEX (4.4 × 10^−117^). The 30 kb indel esv2666691 as a cis-eQTL for *APOBEC3B* is conspicuous since the deletion completely eliminates the coding region of *APOBEC3B*^34^. Therefore, the indel esv2666691, which can cause gene deletion, is pinpointed as the causal variant for the association SNP rs12628403. Collectively, we demonstrate that PExFInS is able to effectively identify variants, especially indels, to tag GWAS SNPs.

## Discussion

GWAS can simultaneously test millions of SNPs in association with human diseases or clinical traits. However, the functional relevance is elusive for the vast majority of the association variants. The causal variant(s) contributing to the disease cannot be directly inferred from the association SNPs. eQTL mapping has become a powerful tool to interpret GWAS findings and facilitate the identification of functional variants or causal genes. In this study, we investigated the expression-correlated variants, including indel cis-eQTLs and SNP cis-eQTLs, in 423 LCLs from six global populations using the 1 KG data of the phase 1 release^20^. There are a total of 21,841 indel cis-eQTLs and 228,743 SNP cis-eQTLs in at least one population under the cutoff *P* value < 10^−4^. A LCL cis-eQTL database has been established and incorporated in the in-house generated pipeline PExFInS. We characterized these cis-eQTLs in the genomic context and demonstrated that cis-eQTLs are enriched around the TSS. Our results indicated that LCL cis-eQTLs represent around one third of NHGRI cataloged GWAS SNPs for more than three quarters of studied diseases and traits. Additionally, the LCL indel cis-eQTLs fundamentally contribute to the GWAS association signals. Likewise, incorporating other cis-eQTL data via PExFInS could result in even greater representation of GWAS SNPs.

The 423 LCLs for cis-eQTL mapping in this study are a subset of 726 LCLs utilized in a previous eQTL study conducted by Stranger *et al.*^13^, since the NGS genotyping data of these 423 LCLs are available in the 1 KG data of phase 1 release^20^. Our cis-eQTL analyses have several merits in comparison with Stranger’s study. Firstly, the initial pool of tested variants in this study is at least 10-time greater than that used in the Stranger’s study. The NGS genotyping data used for this study comprise 38.2 M SNPs and 3.9 M short indels. Consequently, our analysis covered variants with more depth and generated a larger amount of cis-eQTLs, including 228,743 SNP cis-eQTLs and 21,841 indel cis-eQTLs with cis-association *P* value < 10^−4^. Secondly, searching for functional variants in our LCL cis-eQTL database is time-effective, in contrast with the traditional approach in which imputation is required to obtain genotypes for the high LD variants. When a GWAS SNP is input in our LCL cis-eQTL database, we can directly retrieve genotypes of its high LD variants as well as their cis-eQTL data. Thirdly, in the previous study, a single Spearman Rank Correlation model was utilized to test the correlation between the genotypes of each SNP and the expression levels of the corresponding cis gene. The expression-correlated SNPs that do not fit the Spearman Rank Correlation model may not be uncovered. Instead, we performed the genotype-expression correlation analysis with five different models. The smallest correlation *P* value among the five models was designated to each cis-eQTL. This strategy may be able to capture more cis-association signals between variants and expression levels of the correlated genes. Fourthly, as the second largest group of variations in human genome, indels are examined in our study but not in the previous study. We identified and characterized a wealth of expression-correlated indel cis-eQTLs, which might be more important than SNP cis-eQTLs for the regulation of gene expression. The identification of these indel cis-eQTLs may facilitate our understanding towards the molecular mechanisms of human diseases.

Apart from the comprehensive LCL cis-eQTL database, we also created a pipeline PExFInS to fine map disease association loci and pinpoint the potential functional variant(s) tagging one or multiple GWAS SNP(s). In PExFInS, we integrate three categories of analysis, including LD pattern visualization with genotyping data from the 1 KG data of phase 1 release or phase 3 release, regulatory feature mapping with data from Ensembl and UCSC, and eQTL mapping with our newly-generated LCL cis-eQTL data and other published cis-eQTL datasets. Each of these analyses has unique advantages and can supplement with each other. Specifically, LD pattern visualization can be conducted with the 1 KG data of the phase 1 release or phase 3 release. These deep sequencing data can relieve the necessity to conduct more targeted resequencing to identify the potential causal variants tagging GWAS SNPs. Regulatory feature mapping with data from Ensembl and UCSC can directly retrieve evidence of the possible functional consequence of candidate variants. Additionally, eQTL mapping is conducive to prioritize the candidate gene correlated to the GWAS SNPs. PExFInS has been applied to identify several human host factors involved in the pathogenesis of human influenza, including *CD55*^33^, surfactant protein B (*SFTPB*)^35^, galectin 1 (*LGALS1*)^36^ and transmembrane protease, serine 2 (*TMPRSS2*)^37^. Therefore, PExFInS is a powerful tool to interpret the GWAS association signals, extend the GWAS discovery and move toward the biological and mechanistic understanding of human diseases and traits.

## Methods

### cis-eQTL analysis in LCLs from six populations

A total of 423 LCLs across six populations were applied to cis-eQTL analysis. The genotyping data of these LCLs were retrieved from the 1 KG data of phase 1 release. Their corresponding microarray expression data were downloaded from ArrayExpress^38^ with the accession no. E-MTAB-264 and E-MTAB-198. Plink^39^ was utilized to test the association of all variants (MAF >1% and missing rate <20%) residing in 200 kb upstream or downstream region of each RefSeq gene with the expression levels of their corresponding transcript probes. Five different association models were tested, including ADD (multiplicative model or genotypic model testing additivity), GENO_2DF (genotypic model), DOMDEV (genotypic model testing deviation from additivity), DOM (dominant model) and REC (recessive model)^40^. Each cis-eQTL was defined based on the model with the smallest association *P* value among the five test models. cis-eQTLs passed the cutoff *P* value < 10^−2^ were incorporated in the LCL cis-eQTL database.

### Distribution analysis of cis-eQTLs

The cis-eQTLs shared among populations were illustrated with six-way Venn diagrams. In cis-eQTL analysis, each RefSeq gene may be represented by more than one transcript probe in gene expression microarray. Therefore, in order to have an accurate comparison among populations, cis-eQTL is referred to the variant-probe pair. Based on the location in various regions of the genome, cis-eQTLs were grouped into six categories, including ±10 kb to TSS, intron, intergenic region, exon, ncRNA, and 3′UTR. In order to have the distribution of cis-eQTLs under null hypothesis of no true association in these genomic regions, genome-wide permutation was utilized to generate fake cis-eQTLs with Plink. We performed genome-wide permutation 100 times, with each permutation performed by keeping the genotypes together, but swapping each gene expression phenotype. In this setting, only the phenotype-genotype relationship was altered by permutation, while LD patterns between variants remained the same. We assigned the smallest association *P* value among the five test models to each variant, selected fake cis-eQTLs in each permutation dataset with cutoff *P* value < 10^−4^ and pooled these fake cis-eQTLs together. The chi-squared test was utilized to compare the genomic distribution of population-shared and population-specific cis-eQTLs, including indel cis-eQTLs and SNP cis-eQTLs, with the permutation-generated counterparts.

### Analysis of GWAS SNPs in the LCL cis-eQTL database

Totally, 14,718 unique GWAS SNPs with dbSNP rs IDs are associated with 1,099 diseases or traits in the NHGRI cataloged GWAS (released on Feb 10, 2015)^1^. Among these GWAS SNPs, a total of 14,172 SNPs (96.3%) had genotyping data in the 1 KG data of phase 1 release. We searched for these GWAS SNPs in our LCL cis-eQTL database. We also utilized Plink to obtain all high LD (r^2^ ≥ 0.7) indel cis-eQTLs with these GWAS SNPs in a 1000 kb window.

### Development of PExFInS

We created a pipeline, PExFInS, to identify the potential functional indels and SNPs for the user-queried SNPs through the incorporated LD analysis, cis-eQTL mapping and functional annotation. PExFInS can map high LD variants to Ensembl regulatory regions stored in Ensembl Regulatory Build 71 and run ANNOVAR to annotate these high LD variants with annotation databases from UCSC Genome Browser. The pipeline was implemented in SAS language with seven SAS macros working together. BASE SAS software is required to run these SAS macros. VCFtools^41^ was utilized to transform all biallelic variants (MAF >0.1% and missing rate <20%) included in the VCF files of the 1 KG data of phase 1 release (1,092 individuals) and phase 3 release (2,504 individuals) into Plink BED files. All these Plink BED files are included in our pipeline PExFInS. Thus PExFInS can utilize these genotyping data and run Plink and Haploview^30^ to calculate pairwise LD values (r^2^ and D’) and visualize LD pattern among query variants, including indels. PExFInS is freely available at http://sourceforge.net/projects/pexfins/.

## Additional Information

**How to cite this article**: Cheng, Z. *et al.* PExFInS: An Integrative Post-GWAS Explorer for Functional Indels and SNPs. *Sci. Rep.* **5**, 17302; doi: 10.1038/srep17302 (2015).

## Supplementary Material

Supplementary Information

## Figures and Tables

**Table 1:**
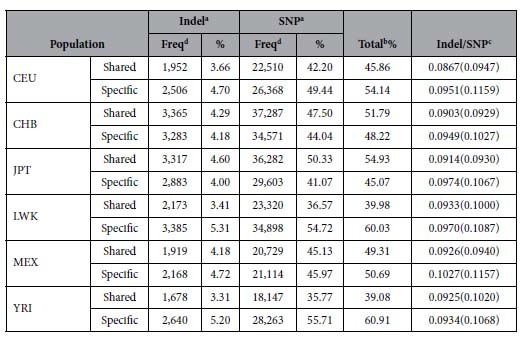
Distribution of population-specific and population-shared LCL cis-eQTLs in six global populations.

^a^The cutoff *P* value of LCL cis-eQTLs is set at <10^−4^.

^b^Percentage of population-shared cis-eQTLs and population-specific cis-eQTLs. The percentages of population-shared cis-eQTLs and population-specific cis-eQTLs among six populations are 46.8 ± 6.4% and 53.18 ± 6.39%, respectively.

^c^Indel/SNP represents the ratio of percentage of indel cis-eQTLs versus that of SNP cis-eQTLs. Ratios of the peak indel versus SNP cis-eQTL of each gene probe are shown in bracket.

^d^Frequency of cis-eQTLs.

## References

[b1] Welter D. *et al.* The NHGRI GWAS Catalog, a curated resource of SNP-trait associations. Nucleic Acids Res. 42, D1001–D1006 (2014). 24316577 10.1093/nar/gkt1229 PMC3965119

[b2] Altshuler D. *et al.* A map of human genome variation from population-scale sequencing. Nature 467, 1061–1073 (2010). 20981092 10.1038/nature09534 PMC3042601

[b3] Zerbino D. R., Wilder S. P., Johnson N., Juettemann T. & Flicek P. R. The ensembl regulatory build. Genome Biol. 16, 56 (2015). 25887522 10.1186/s13059-015-0621-5 PMC4407537

[b4] Johnson A. D. *et al.* SNAP: a web-based tool for identification and annotation of proxy SNPs using HapMap. Bioinformatics 24, 2938–2939 (2008). 18974171 10.1093/bioinformatics/btn564 PMC2720775

[b5] Pruim R. J. *et al.* LocusZoom: regional visualization of genome-wide association scan results. Bioinformatics 26, 2336–2337 (2010). 20634204 10.1093/bioinformatics/btq419 PMC2935401

[b6] Mills R. E. *et al.* Natural genetic variation caused by small insertions and deletions in the human genome. Genome Res. 21, 830–839 (2011). 21460062 10.1101/gr.115907.110 PMC3106316

[b7] Karban A. S. *et al.* Functional annotation of a novel NFKB1 promoter polymorphism that increases risk for ulcerative colitis. Hum. Mol. Genet. 13, 35–45 (2004). 14613970 10.1093/hmg/ddh008

[b8] Lin S. C. *et al.* Correlation between functional genotypes in the matrix metalloproteinases-1 promoter and risk of oral squamous cell carcinomas. J. Oral Pathol. Med. 33, 323–326 (2004). 15200479 10.1111/j.1600-0714.2004.00214.x

[b9] Bhangale T. R., Rieder M. J., Livingston R. J. & Nickerson D. A. Comprehensive identification and characterization of diallelic insertion-deletion polymorphisms in 330 human candidate genes. Hum. Mol. Genet. 14, 59–69 (2005). 15525656 10.1093/hmg/ddi006

[b10] Clark T. G. *et al.* Functional constraint and small insertions and deletions in the ENCODE regions of the human genome. Genome Biol. 8, R180 (2007). 17784950 10.1186/gb-2007-8-9-r180 PMC2375018

[b11] Hu J. & Ng P. C. Predicting the effects of frameshifting indels. Genome Biol. 13 (2012). 10.1186/gb-2012-13-2-r9 PMC3334572 22322200

[b12] Yang T. P. *et al.* Genevar: a database and Java application for the analysis and visualization of SNP-gene associations in eQTL studies. Bioinformatics 26, 2474–2476 (2010). 20702402 10.1093/bioinformatics/btq452 PMC2944204

[b13] Stranger B. E. *et al.* Patterns of cis regulatory variation in diverse human populations. PLoS Genet. 8, e1002639 (2012). 22532805 10.1371/journal.pgen.1002639 PMC3330104

[b14] Innocenti F. *et al.* Identification, replication, and functional fine-mapping of expression quantitative trait loci in primary human liver tissue. PLoS Genet. 7(5), e1002078 (2011). 21637794 10.1371/journal.pgen.1002078 PMC3102751

[b15] Hulse A. M. & Cai J. J. Genetic variants contribute to gene expression variability in humans. Genetics 193, 95–108 (2013). 23150607 10.1534/genetics.112.146779 PMC3527258

[b16] Fu J. Y. *et al.* Unraveling the regulatory mechanisms underlying tissue-dependent genetic variation of gene expression. PLoS Genet. 8(1), e1002431 (2012). 22275870 10.1371/journal.pgen.1002431 PMC3261927

[b17] Chen L., Page G. P., Mehta T., Feng R. & Cui X. Q. Single nucleotide polymorphisms affect both cis- and trans-eQTLs. Genomics 93, 501–508 (2009). 19248827 10.1016/j.ygeno.2009.01.011 PMC4041081

[b18] Bushel P. R. *et al.* Population differences in transcript-regulator expression quantitative trait loci. PLoS One 7(3), e34286 (2012). 22479588 10.1371/journal.pone.0034286 PMC3313997

[b19] Montgomery S. B. *et al.* The origin, evolution, and functional impact of short insertion-deletion variants identified in 179 human genomes. Genome Res. 23, 749–761 (2013). 23478400 10.1101/gr.148718.112 PMC3638132

[b20] Abecasis G. R. *et al.* An integrated map of genetic variation from 1,092 human genomes. Nature 491, 56–65 (2012). 23128226 10.1038/nature11632 PMC3498066

[b21] Nicolae D. L. *et al.* Trait-associated SNPs are more likely to be eQTLs: annotation to enhance discovery from GWAS. PLoS Genet. 6, e1000888 (2010). 20369019 10.1371/journal.pgen.1000888 PMC2848547

[b22] Lappalainen T. *et al.* Transcriptome and genome sequencing uncovers functional variation in humans. Nature 501, 506–511 (2013). 24037378 10.1038/nature12531 PMC3918453

[b23] Huang J. *et al.* eQTL mapping identifies insertion- and deletion-specific eQTLs in multiple tissues. Nat Commun 6, 6821 (2015). 25951796 10.1038/ncomms7821 PMC4929061

[b24] Hao K. *et al.* Lung eQTLs to help reveal the molecular underpinnings of asthma. PLoS Genet. 8, e1003029 (2012). 23209423 10.1371/journal.pgen.1003029 PMC3510026

[b25] Fairfax B. P. *et al.* Innate immune activity conditions the effect of regulatory variants upon monocyte gene expression. Science 343, 1118-+ (2014). 10.1126/science.1246949 PMC4064786 24604202

[b26] Lee M. N. *et al.* Common genetic variants modulate pathogen-sensing responses in human dendritic cells. Science 343, 1246980 (2014). 24604203 10.1126/science.1246980 PMC4124741

[b27] Zhang X. *et al.* Identification of common genetic variants controlling transcript isoform variation in human whole blood. Nat. Genet. 47(4), 347–52 (2015). 10.1038/ng.3220 PMC8273720 25685889

[b28] Battle A. *et al.* Impact of regulatory variation from RNA to protein. Science 347, 664–667 (2015). 25657249 10.1126/science.1260793 PMC4507520

[b29] Wu L. *et al.* Variation and genetic control of protein abundance in humans. Nature 499, 79–82 (2013). 23676674 10.1038/nature12223 PMC3789121

[b30] Barrett J. C., Fry B., Maller J. & Daly M. J. Haploview: analysis and visualization of LD and haplotype maps. Bioinformatics 21, 263–265 (2005). 15297300 10.1093/bioinformatics/bth457

[b31] Wang K., Li M. & Hakonarson H. ANNOVAR: functional annotation of genetic variants from high-throughput sequencing data. Nucleic Acids Res. 38, e164 (2010). 20601685 10.1093/nar/gkq603 PMC2938201

[b32] Kent W. J. *et al.* The human genome browser at UCSC. Genome Res. 12, 996–1006 (2002). 12045153 10.1101/gr.229102 PMC186604

[b33] Zhou J. *et al.* A functional variation in CD55 increases the severity of 2009 pandemic H1N1 influenza A virus infection. J. Infect. Dis. 206, 495–503 (2012). 22693232 10.1093/infdis/jis378

[b34] Long J. R. *et al.* A common deletion in the APOBEC3 genes and breast cancer risk. Jnci-Journal of the National Cancer Institute 105, 573–579 (2013). 10.1093/jnci/djt018 PMC3627644 23411593

[b35] To K. K. *et al.* Surfactant protein B gene polymorphism is associated with severe influenza. Chest 145, 1237–1243 (2014). 24337193 10.1378/chest.13-1651

[b36] Chen Y. *et al.* Functional variants regulating LGALS1 (Galectin 1) expression affect human susceptibility to influenza A(H7N9). Sci. Rep. 5, 8517 (2015). 25687228 10.1038/srep08517 PMC4649671

[b37] Cheng Z. *et al.* Identification of TMPRSS2 as a susceptibility gene for severe 2009 pandemic A(H1N1) influenza and A(H7N9) influenza. J. Infect. Dis., 212(8), 1214–21 (2015). 25904605 10.1093/infdis/jiv246 PMC7107393

[b38] Parkinson H. *et al.* ArrayExpress update-from an archive of functional genomics experiments to the atlas of gene expression. Nucleic Acids Res. 37, D868–D872 (2009). 19015125 10.1093/nar/gkn889 PMC2686529

[b39] Purcell S. *et al.* PLINK: A tool set for whole-genome association and population-based linkage analyses. Am. J. Hum. Genet. 81, 559–575 (2007). 17701901 10.1086/519795 PMC1950838

[b40] Clarke G. M. *et al.* Basic statistical analysis in genetic case-control studies. Nat. Protoc. 6, 121–133 (2011). 21293453 10.1038/nprot.2010.182 PMC3154648

[b41] Danecek P. *et al.* The variant call format and VCFtools. Bioinformatics 27, 2156–2158 (2011). 21653522 10.1093/bioinformatics/btr330 PMC3137218

