# Supplementary material for: PExFInS: An Integrative Post-GWAS Explorer for Functional Indels and SNPs
Source: Sci Rep. 2015 Nov 27;5:17302. doi: 10.1038/srep17302 (PMC4661514; doi:10.1038/srep17302)

PExFInS: An Integrative Post-GWAS Explorer for Functional Indels and SNPs

Zhongshan Cheng[1], Hin Chu[1,2,3,4], Yanhui Fan[5,6], Cun Li[1], You-Qiang Song[5], Jie Zhou*[1,2,3,4], Kwok-Yung Yuen[1,2,3,4]

Affiliations: [1] Department of Microbiology; [2] State Key Laboratory of Emerging Infectious Diseases; [3] Research Centre of Infection and Immunology; [4] Carol Yu Centre for Infection; [5] Department of Biochemistry; [6] Center for Genomic Sciences; The University of Hong Kong, Hong Kong Special Administrative Region, China.

*Correspondence: Jie Zhou (jiezhou@hku.hk) Department of Microbiology, The University of Hong Kong, Queen Mary Hospital, Pokfulam Road, Hong Kong Special Administrative Region, China.

Supplementary Fig. 1. The distribution of lymphoblastoid cell line (LCL) cis-eQTLs with six-way Venn diagram. cis-eQTLs are defined as SNP/indel-probe pairs around 200kb upstream or downstream of each RefSeq gene. Indel cis-eQTLs and SNP cis-eQTLs in LCLs are analyzed in six global populations (CHB, JPT, CEU, YRI, LWK and MEX). At cis-eQTL *P* value $<10^{-4}$, the numbers of cis-eQTLs shared in only two populations are highlighted with red. The numbers of population-specific cis-eQTLs and those shared in more than 2 populations are also illustrated in the figure. The numbers of SNP and indel cis-eQTLs present in all investigated populations are 3,743 and 333, respectively. The total numbers of indel cis-eQTLs and SNP cis-eQTLs in at least one population are also presented at the upper-left corner of each plot.

Supplementary Fig. 1

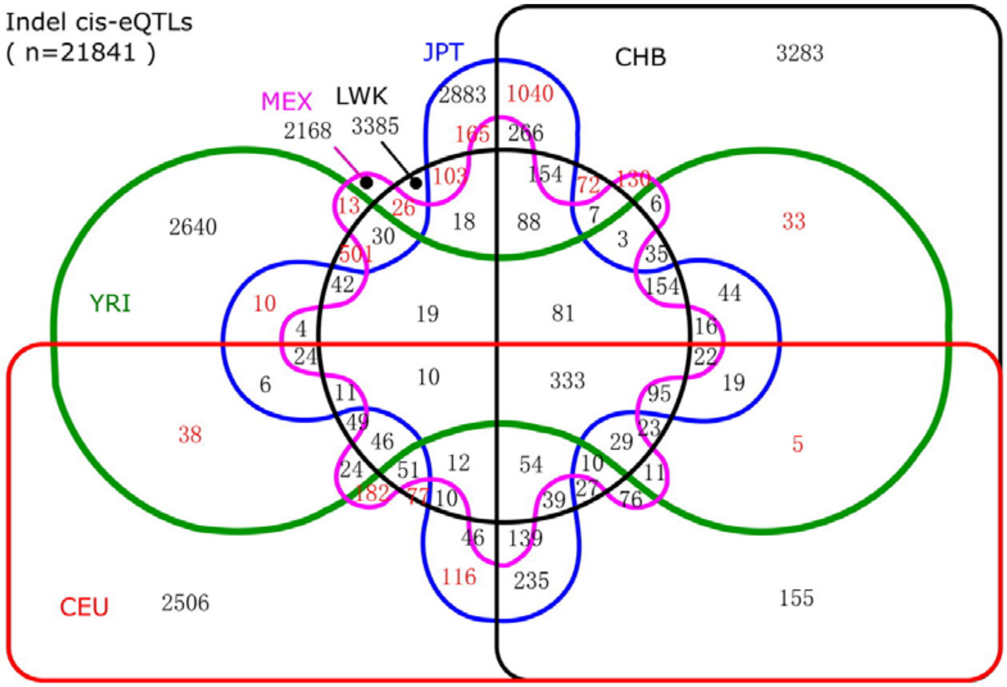

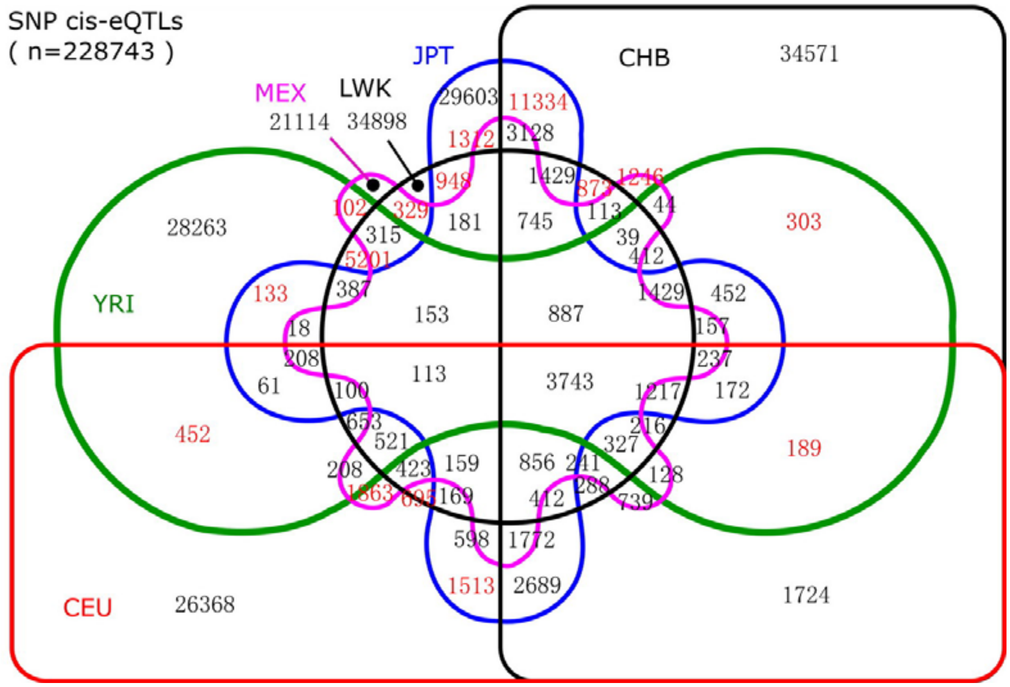

Supplement: Supplementary Information [file srep17302-s1.pdf]
